# Deficits in spontaneous and stimulus-dependent retrieval as an early sign of abnormal aging

**DOI:** 10.1038/s41598-022-13745-6

**Published:** 2022-06-10

**Authors:** Michał Wereszczyński, Agnieszka Niedźwieńska

**Affiliations:** Department of Psychology, Jagiellonian University, Kraków, Poland

**Keywords:** Psychology, Human behaviour, Geriatrics, Dementia

## Abstract

Research on early cognitive markers of Alzheimer’s disease is primarily focused on episodic memory tests that involve deliberate retrieval. Our purpose was to provide clear evidence to support a novel Spontaneous Retrieval Deficit hypothesis, which predicts that people at pre-clinical stages of dementia, including those with amnestic Mild Cognitive Impairment (aMCI), are particularly impaired on tasks based on spontaneous retrieval. We compared 27 aMCI individuals and 27 healthy controls on mind-wandering while performing a task during which there were exposed to either highly meaningful or unmeaningful pictures. The substantial reduction in mind-wandering among individuals with aMCI was found with exposure to highly meaningful stimuli, but not to unmeaningful pictures, and it was most pronounced for past-oriented thoughts, i.e., involuntary autobiographical memories. Those findings provide strong support for this novel hypothesis, and show that it is the spontaneous, but bottom-up and cue-driven processes, for which meaningful environmental stimuli are crucial, that are very promising early markers of the disease.

## Introduction

With increased life expectancy, the number of older adults diagnosed with Alzheimer’s disease (AD) continues to grow^1^. Impairment of declarative memory, one of the key symptoms indicating AD, is associated with cerebral pathological changes which may start years, or even decades, before the clinical diagnosis of dementia^2,3^. In the absence of effective drug treatment, research has increasingly focused on identifying individuals most at risk of developing AD who could most likely benefit from early disease management and care^4^. One such group are those with various forms of Mild Cognitive Impairment, the most prevalent being amnestic MCI (aMCI). It manifests in subjective and objective memory deficits, as evidenced by the performance on episodic memory tests, without the loss of functional independence that is characteristic of AD^4^. Individuals with aMCI have increased yearly conversion rates to AD (10–15%) and are more likely than normally aging adults to have brain pathology characteristic of AD^3,5^.

A novel hypothesis has been recently formulated which stipulates that spontaneous (i.e., unintentional and effortless) retrieval processes, which are generally preserved in healthy aging^6–8^, will be significantly compromised in individuals with aMCI, and at the earliest stages of AD^9^. It also argues^9^ that cognitive tasks that are based on spontaneous retrieval may be more sensitive to early cognitive deterioration than tasks that rely on deliberate and effortful encoding and retrieval. Deliberate processes are mediated by executive and attentional control regions in the prefrontal cortex which become substantially compromised at later stages of AD. Since all currently used neuropsychological tests of episodic memory rely on deliberate memory processes, the hypothesis, if confirmed, can transform the current theoretical understanding of the most effective early cognitive markers of the disease.

The spontaneous retrieval deficit hypothesis (the SRD hypothesis) is based on the results of the neuroimaging studies showing that, during the etiology of AD, neurological structures of the Default Mode Network (DMN) tend to degenerate much earlier than other parts of the central nervous system (see ^9^, for a review of evidence). The first signs of neuropathological changes within AD tend to occur in posterior parts of the cortex, with the anterior and dorsolateral prefrontal cortex remaining relatively intact^10,11^, resulting in disproportionate temporoparietal atrophy in the early stages of the disease^12,13^. The pathology involves the accumulation of tau-positive neurofibrillary tangles in medial temporal lobe structures, spreading from the entorhinal cortex to the hippocampus^14^, and the formation of β-amyloid plaques in the medial prefrontal and posteromedial cortices, especially in the posterior cingulate cortex and adjacent areas^15,16^. These neuropathological processes, especially β-amyloid accumulation, may progress insidiously, for many years, along a slow pre-symptomatic course before clinical symptoms are evident^4^.

Importantly, the posterior cingulate cortex, medial temporal lobe, and medial prefrontal cortex are anatomically and functionally strongly interconnected and form part of the DMN^17,18^. DMN activity has been traditionally linked to mind-wandering, which involves spontaneous shifts of attention from the external world to one’s inner thoughts^19,20^. Links between mind-wandering and increased DMN activity have also been demonstrated in several fMRI studies (see^9^, for a review of evidence). Mind-wandering share similar characteristics with several other phenomena of spontaneous cognitions such as, for example, involuntary autobiographical memories^21,22^, or those aspects of prospective memory that involve effortlessly remembering previously intended actions in response to a particular target event^23,24^. What these phenomena share with mind-wandering episodes is that thoughts and memories come to mind spontaneously and effortlessly, without any deliberate intention to think about them.

A few recent behavioural studies support the SRD hypothesis by showing the deficit of spontaneous retrieval in individuals with aMCI, and at early stages of AD, in prospective memory^25–27^ and mind-wandering^28,29^. Niedźwieńska and Kvavilashvili^28^ used thought probes alongside an easy vigilance task, during which cue phrases, irrelevant to the ongoing task, were frequently presented. Participants with aMCI reported significantly fewer spontaneous task-unrelated thoughts than healthy older adults, especially thoughts about past (i.e., involuntary autobiographical memories). Importantly, the vast majority of spontaneous thoughts were triggered by irrelevant cue phrases. The decrease in the frequency of task-unrelated thoughts were also found among patients with very mild to mild AD, as compared with healthy controls, while performing the Sustained Attention to Response Task, with thought probes^29^.

However, two behavioral studies did not find support for the SRD hypothesis^30,31^. Patients with probable AD and healthy controls did not differ in the frequency of on- and off-task thoughts reported during a shape expectations task^30^. However, by contrast to^28^, this study also did not examine whether participants’ off-task thoughts were spontaneous or intentional, and, second, participants were not exposed to meaningful stimuli during the ongoing task. With regard to the first issue, it has been shown that participants report engaging in task-unrelated thoughts deliberately^8,32,33^, and therefore not all task-unrelated thoughts qualify as spontaneous cognitions. As to the second issue, an important distinction between stimulus-independent and stimulus-dependent mind-wandering has been proposed^9,34^: i.e., thoughts may occur without any noticeable trigger or pop into mind in response to a cue which could be an incidental stimulus in the external environment. Distinguishing stimulus-dependent mind-wandering is supported by empirical evidence showing that when participants are exposed to meaningful incidental stimuli, stimulus-dependent spontaneous thoughts is the norm rather than the exception, both in the laboratory^32^ and in everyday life^8^. FMRI studies also show that the posterior cingulate cortex, a key hub of the DMN, is crucially involved in the manifestation of spontaneous thoughts in response to stimuli encountered in the environment^35–37^. Based on the distinction between stimulus-independent and stimulus-dependent mind-wandering, the SRD hypothesis stipulates that aMCI and very mild AD primarily penalise spontaneous, but bottom-up and cue-driven, retrieval processes for which the presence of meaningful cues is essential^9^ (see also^32^).

The other study, which did not find support for the SRD hypothesis, asked participants to watch an audio-visual material which presented common activities, famous actors and popular songs from the period corresponding to participants’ youth^31^. The experimenter recorded participants’ commentaries that they were making, unprompted, during the film and briefly after that. Although participants with mild to moderate AD shared more commentaries relating to autobiographical memories, as compared with healthy controls, again the experimenter did not examine whether their commentaries were based on thoughts that had entered their mind spontaneously or whether they had deliberately decided to think about them. Importantly, in contrast to all previous studies that examined mind-wandering in aMCI and mild AD by using thought probes^28,29^, participants were not systematically asked to reveal their thoughts. Therefore, the study may have measured inhibitory control rather than spontaneous retrieval efficiency, and a lower number of commentaries in healthy controls may have reflected a greater reluctance to share their thoughts. This explanation is partially in line with the authors’ suggestion that deficits in inhibitory control made patients less able to hold back emotion-expressive behavior when being exposed to emotionally arousing material^31^.

Therefore, we suggest that discrepant findings regarding the SRD hypothesis may be due to the fact that previous studies have used tasks that, to varying degrees, meet the criteria that are necessary to capture group differences in spontaneous and cue-driven retrieval processes. First, the ongoing task difficulty needs to be low and matched between patients and healthy controls to exclude the possibility that cognitive resources, which could be disposed for spontaneous processes, will be much more limited among patients. Second, there should be stimuli in the environment that have the potential to serve as cues to trigger spontaneous thoughts. Third, the experimenter needs to distinguish between spontaneous and intentional task-unrelated thoughts. Fourth, thought probes need to be used during which participants are directly asked what they were thinking about, to avoid the impact of group differences in reluctance to share inner thoughts.

The first goal of our study was to test the SRD hypothesis while using a task that meets all the necessary criteria to capture spontaneous and cue-driven retrieval processes. Second, we tested the robustness of the spontaneous retrieval deficits by investigating whether they generalise to situations when individuals are exposed to potential cues that are different in their nature to the ones used so far. Since behavioral evidence for the SRD hypothesis comes from the studies that used verbal^28^ or digital^29^ stimuli, we used pictures as potential cues. Third, to test the prediction that it is spontaneous, but bottom-up and cue-driven, retrieval processes that are impaired in aMCI, and that it is meaningful cues that are essential for eliciting such processes, we investigated, for the first time, whether highly meaningful stimuli would better enable to demonstrate spontaneous retrieval deficits in aMCI, compared to unmeaningful stimuli.

To accomplish these goals, we used a task of distinguishing between natural and man-made objects, visually presented^6^, with participants with aMCI and matched healthy older controls. Both groups were given thought probes. We developed two versions of the task: one with highly meaningful pictures, i.e., rated by participants as highly familiar based on their personal experience, and the other version with unmeaningful pictures, i.e., rated by participants as highly unfamiliar based on their personal experience. Strictly speaking, unmeaningful objects were not completely unknown to participants, but, judging by the familiarity ratings, they had not been present, or had been present very rarely, in participants’ individual past, and therefore had no, or very little, personal meaning to them.

In line with the SRD hypothesis, we expected that aMCI participants would report significantly fewer spontaneous task-unrelated thoughts than healthy controls (HC). We also expected that the reduction of spontaneous thoughts in aMCI would manifest more strongly with exposure to highly meaningful stimuli, compared to exposure to unmeaningful stimuli. Based on^28^, we also expected thoughts about past, compared to thoughts about future and present, to most strongly demonstrate the reduction of spontaneous thoughts in aMCI.

## Results

The alpha level adopted for determining significance of the results was set at 0.05. The effect size was measured by partial eta squared, η_p_^2^ (small 0.01, medium 0.06, large 0.16) or Cohen’s d (small 0.20, medium 0.50, large 0.80)^38^.

### Types of responses recorded during the task

To test the SRD hypothesis, and based on participants’ answers whether they had any thought at a thought probe, and if yes, whether it was related to the experience of doing the Man-made/Natural Task, and whether it was spontaneous or deliberate, we grouped participants’ responses into: (1) spontaneous task-unrelated thoughts; (2) spontaneous task-related thoughts (3) deliberate thoughts; (4) no thoughts. The vast majority of spontaneous task-unrelated thoughts in both groups were stimulus-dependent (91% in aMCI and 81% in HC).

**Figure 1:**
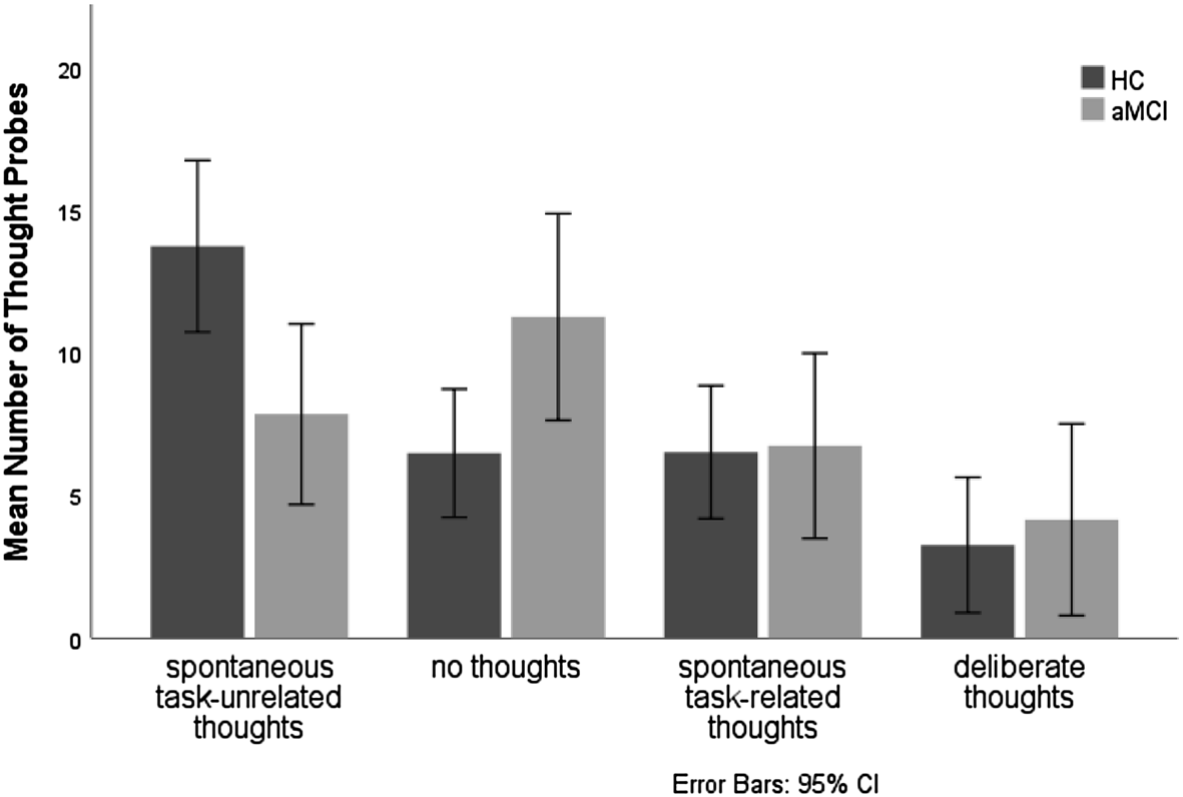
Mean number of thought probes as a function of response type (spontaneous task-related thoughts vs. spontaneous task-unrelated thoughts vs. deliberate thoughts vs. no thoughts) and group (aMCI participants vs. healthy controls).

To assess the hypothesis that aMCI participants would report significantly fewer spontaneous task-unrelated thoughts than HC, the mean number of thought probes in each of the 4 response types (spontaneous task-related, spontaneous task-unrelated, deliberate, no thoughts) were entered into a MANOVA with group (HC vs aMCI) as a between-subject factor. There was a significant main effect of group [*F* (3, 50) = 3.075, *p* = 0.036; η_*p*_^2^ = 0.156] (Fig. 1). As expected, participants with aMCI experienced significantly fewer spontaneous task-unrelated thoughts than HC [*F* (1, 52) = 7.672, *p* = 0.008; η_*p*_^2^ = 0.129]. There were significantly more “no thoughts” probes in aMCI individuals than in HC [*F* (1, 52) = 5.299, *p* = 0.025; η_*p*_^2^ = 0.092]. No other group differences were significant (all group comparisons in “Supplementary Material”).

### Spontaneous task-unrelated thoughts as a function of stimulus type

**Figure 2:**
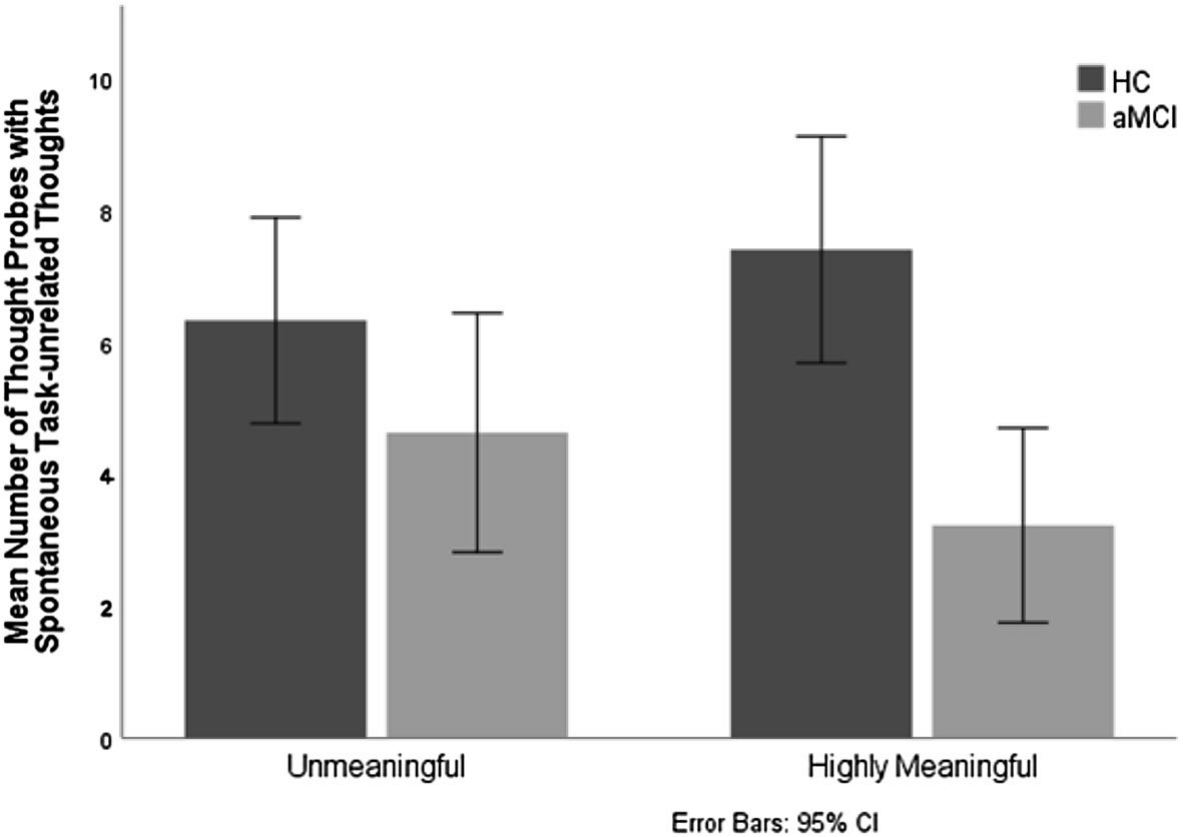
Mean number of thought probes with spontaneous task-unrelated thoughts as a function of stimulus type (highly meaningful vs unmeaningful) and group (aMCI participants vs. healthy controls).

To assess the hypothesis that spontaneous retrieval deficits in the aMCI group would be particularly pronounced with exposure to highly meaningful stimuli, as compared with exposure to unmeaningful stimuli, the mean number of thought probes with spontaneous task-unrelated thoughts was entered into a 2 group (HC vs. aMCI) by 2 stimulus type (highly meaningful vs. unmeaningful) mixed ANOVA with the repeated measure on the second factor. There was a significant main effect of group [*F* (1, 52) = 7.678, *p* = *0.0*08; η_*p*_^2^ = 0.129], and a significant group by stimulus type interaction [*F* (1, 52) = 9.728, *p* = *0.0*03; η_*p*_^2^ = 0.158] (Fig. 2). As predicted, aMCI participants reported significantly fewer spontaneous task-unrelated thoughts than HC when exposed to highly meaningful stimuli [*F* (1, 52) = 14.412, *p* = *0.0*00; η_*p*_^2^ = 0.217], but not when exposed to unmeaningful stimuli [*F* (1, 52) = 2.135, *p* = *0.1*50; η_*p*_^2^ = 0.039]. The number of spontaneous task-unrelated thoughts did not significantly differ for highly meaningful and unmeaningful stimuli in HC [*F* (1, 52) = 3.645, *p* = *0.0*62; η_*p*_^2^ = 0.066], but aMCI participants had more spontaneous task-unrelated thoughts for unmeaningful stimuli compared to highly meaningful stimuli [*F* (1, 52) = 6.258, *p* = *0.0*16; η_*p*_^2^ = 0.107] (all comparisons in “Supplementary Material”).

### Spontaneous task-unrelated thoughts as a function of thought temporality

**Figure 3:**
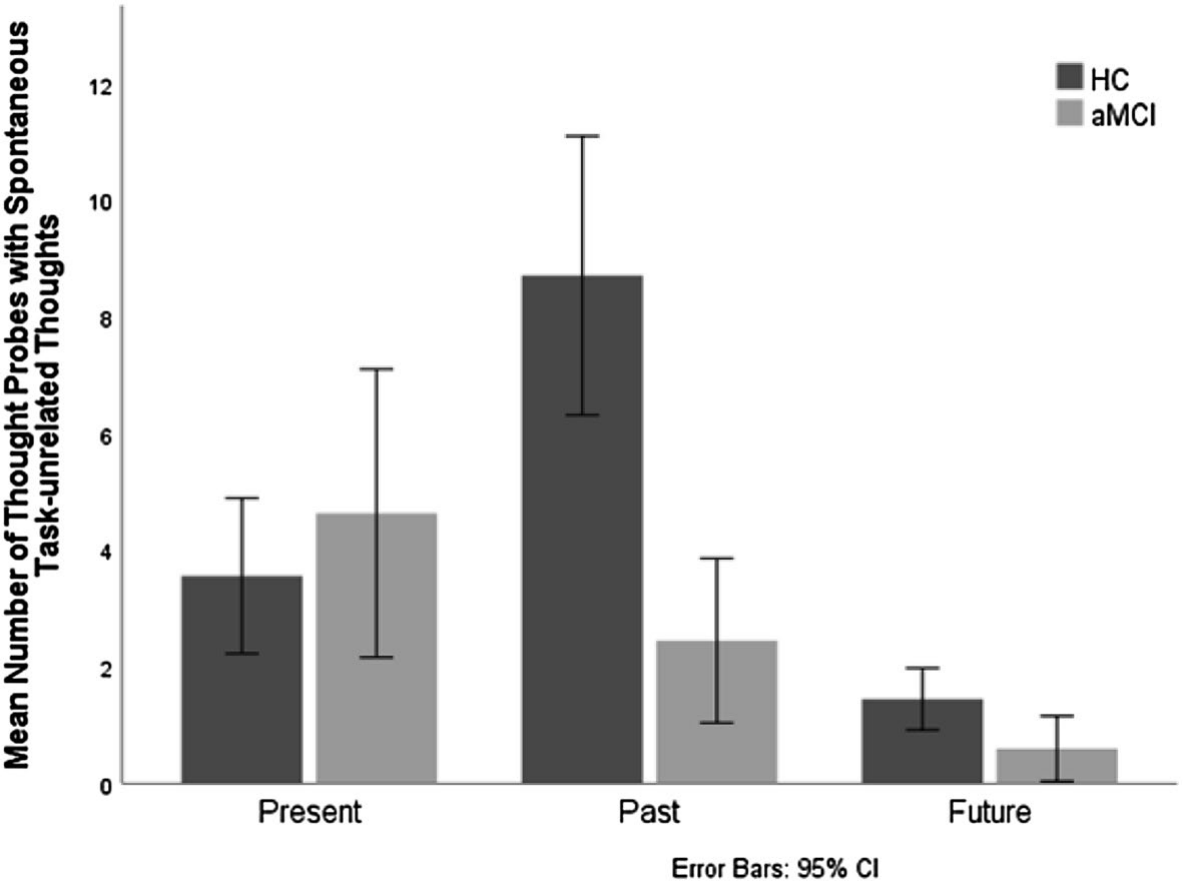
Mean number of thought probes with spontaneous task-unrelated thoughts as a function of temporal orientation (present vs. past vs. future) and group (aMCI participants vs. healthy controls).

To assess the hypothesis that thoughts about past events (i.e. involuntary autobiographical memories) would most strongly demonstrate the reduction of spontaneous thoughts in participants with aMCI, the mean number of spontaneous task-unrelated thoughts was entered into a 2 group (HC vs. aMCI) by 3 temporal orientation (future vs. past vs. present) mixed ANOVA with the repeated measure on the second factor. There were significant main effects of group [*F* (1, 52) = 8.041, *p* = *0.0*06; η_*p*_^2^ = 0.134] and temporal orientation [*F* (2, 51) = 33.78, *p* = *0.0*00; η_*p*_^2^ = 0.570]. These effects were qualified by a significant group by temporal orientation interaction [*F* (2, 51) = 9.50, *p* = 0.000; η_*p*_^2^ = 0.272] (Fig. 3). As predicted, aMCI participants had significantly fewer past-oriented thoughts than HC [*F* (1, 52) = 21.482, *p* = 0.000; η_*p*_^2^ = 0.292]. This difference was also significant for future-oriented thoughts, with much smaller effect size [*F* (1, 52) = 5.136, *p* = *0.0*28; η_*p*_^2^ = 0.090] (all group comparisons in “Supplementary Material”).

### Past-oriented thoughts (involuntary autobiographical memories) as a function of stimulus type

**Figure 4:**
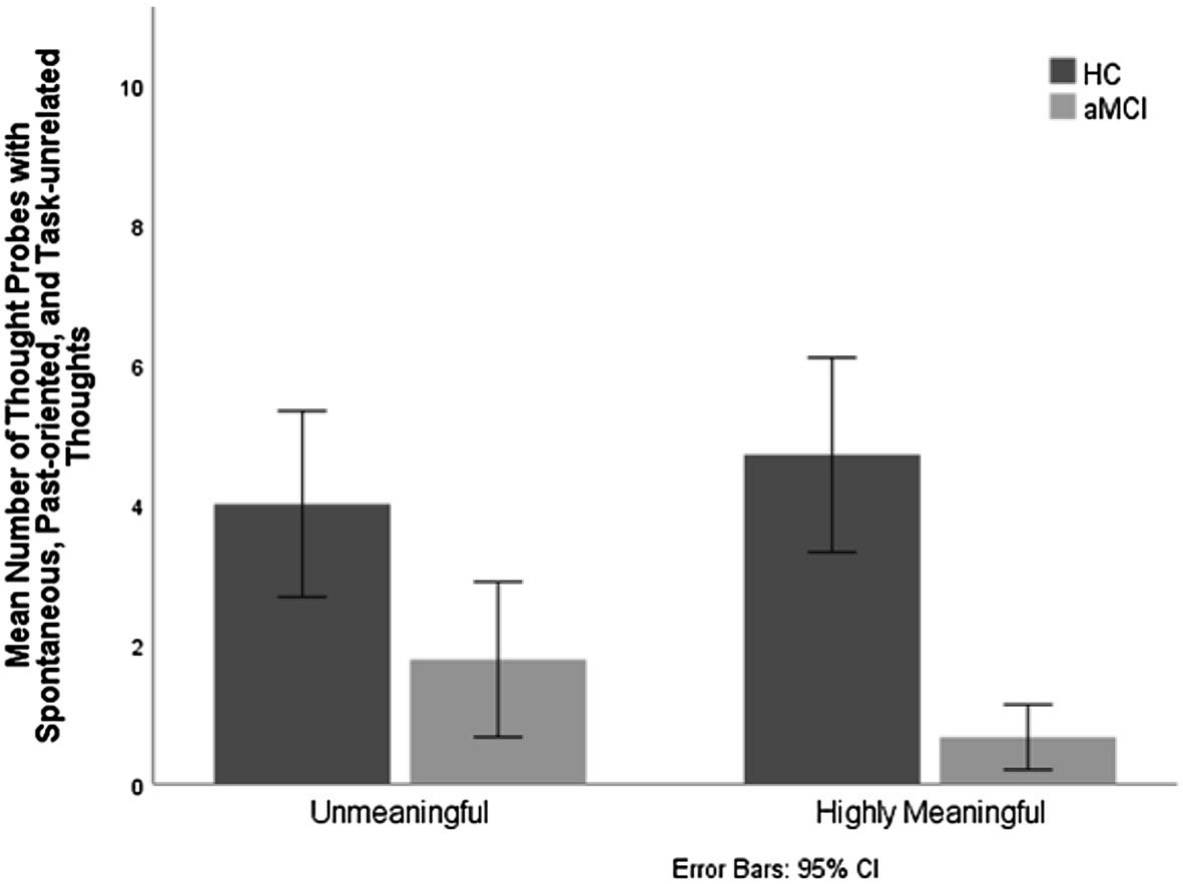
Mean number of thought probes with spontaneous, past-oriented, and task-unrelated thoughts as a function of stimulus type (highly meaningful vs unmeaningful) and group (aMCI participants vs. healthy controls).

Since the reduction of spontaneous task-unrelated thoughts in aMCI was most strongly pronounced in involuntary memories, we conducted additional analyses to investigate whether the quality of stimuli influenced the size of the reduction in memories in the same way as it was predicted, and indeed found, for spontaneous task-unrelated thoughts overall. Therefore, the mean number of thought probes with past-oriented, spontaneous and task-unrelated thoughts was entered into a 2 group (HC vs. aMCI) by 2 stimulus type (highly meaningful vs. unmeaningful) mixed ANOVA with the repeated measure on the second factor. There was a significant main effect of group [*F* (1, 52) = 21.482, *p* = *0.0*00; η_*p*_^2^ = 0.292], and a significant group by stimulus type interaction [*F* (1, 52) = 5.348, *p* = *0.0*25; η_*p*_^2^ = 0.093] (Fig. 4). Individuals with aMCI had significantly fewer past-oriented thoughts than HC for both highly meaningful and unmeaningful stimuli, but, as it could be expected, the effect size of this difference was much bigger for highly meaningful stimuli [*F* (1, 52) = 31.991, *P* = 0.000; η_*p*_^2^ = 0.381] compared to unmeaningful stimuli [*F* (1, 52) = 6.952, *p* = *0.0*11; η_*p*_^2^ = 0.118]. No other effects were significant (all comparisons in “Supplementary Material”).

### Potential confounds to spontaneous retrieval deficits

It may be argued that the Man-made/Natural Task was easier, and therefore more boring, for HC, compared to aMCI individuals, which made them mind-wander more. In a similar vein, the Man-made/Natural Task may have been more difficult for aMCI individuals, and therefore they did not have enough cognitive resources left for spontaneous processes. However, the data indicate otherwise (Table 1). Both groups performed at ceiling on the Man-made/Natural Task, and they did not differ on any of the performance measures (the percentage of correct answers out of all answers provided and mean response time), except for the number of invalid answers caused by pressing the wrong keyboard button or missing an answer. Furthermore, the groups expressed the same level of interest in the task. However, to exclude this alternative explanation of group differences in mind-wandering, we investigated whether the level of performance on the Man-made/Natural Task influenced the pattern of group differences in the number of spontaneous task-unrelated thoughts. The mean number of spontaneous task-unrelated thoughts was entered into a one-way ANCOVA, with group as a between-subject factor and the three measures of performance on the Man-made/Natural Task as covariates. None of the covariates was significant: mean response time [*F* (1, 49) = 0.196; *p* = 0.660; η_*p*_^2^ = 0.004]; number or invalid answers [*F* (1, 49) = 0.915; p = 0.344; η_*p*_^2^ = 0.018]; percentage of correct answers [*F* (1, 49) = 2.702; *p* = 0.107; η_*p*_^2^ = 0.052]. The main effect of group was significant [*F* (1, 49) = 7.003; *p* = 0.011; η_*p*_^2^ = 0.125], such that aMCI participants continued to mind-wander less, after controlling for performance on the Man-made/Natural Task. This speaks against the task difficulty as being a potential confound to spontaneous retrieval deficits.

**Table 1:** Mean (standard deviation) accuracy, response time, invalid answers and interest ratings for the man-made/natural task in participants with aMCI and healthy controls, and results of independent samples T-test.

Man-made/natural task	aMCI (n = 27)^a^	Healthy controls (n = 27)	*t*	*df*	*p*	*d*
Accuracy	0.91 (0.12)	0.96 (0.02)	− 1.94	52	0.057	0.58
Response time (ms)	2096.05 (542.15)	1889.65 (345.41)	1.66	52	0.101	0.45
Invalid answers	32.37 (41.30)**	10.14 (8.14)	− 2.98	52	0.008	0.71
Interest^b^	7.96 (2.08)	6.84 (3.04)	1.54	50	0.129	0.43

*aMCI* amnestic Mild Cognitive Impairment.

Differences between aMCI and HC are indicated by ** p < 0.01.

^a^Except for the interest ratings that were not provided by two participants (one in each group).

^b^Task interest ratings were made on a 10-point scale (1 = very boring; 10 = very interesting).

It may also be argued that the group differences in cognitive functions, other than memory retrieval, may explain less mind-wandering in aMCI. Again, to exclude this alternative explanation, we investigated whether other cognitive functions, as measured by the Addenbrooke’s Cognitive Examination-III (ACE-III), influenced the pattern of group differences in the number of spontaneous task-unrelated thoughts. The mean number of spontaneous task-unrelated thoughts was entered into a one-way ANCOVA, with group as a between-subject factor and a composite score on the ACE-III, which included the attention, fluency, language and visuospatial abilities subscales, as a covariate. The covariate was not significant [*F* (1, 51) = 0.126 *p* = 0.724; η_*p*_^2^ = 0.002]. The main effect of group was significant [*F* (1, 51) = 6.282; *p* = 0.015; η_*p*_^2^ = 0.110], such that aMCI participants continued to mind-wander less, after controlling for performance on the ACE-III. This speaks against the differences in other cognitive functions as being potential confounds to spontaneous retrieval deficits.

The aMCI group and HC did not differ in their ratings of how difficult the task of categorizing thoughts was for them (*p* = 0.265).

## Discussion

A recently formulated SRD hypothesis stipulates that tasks based on spontaneous retrieval are most compromised in aMCI and at early stages of AD, and are better early cognitive markers of the disease, compared to tasks that rely on deliberate episodic memory processes^9^. This hypothesis is highly counterintuitive because it challenges current theories of cognitive aging^39,40^ which predict that both typical and atypical aging predominantly impair performance on difficult cognitive tasks that rely on deliberate and strategic processes. It also speaks against the current practice of the dementia diagnosis which involves neuropsychological tests based on strategic encoding and retrieval processes. However, recent neuropsychological studies have shown that the structures responsible for spontaneous retrieval degenerate much earlier during the dementia development than those mediating strategic memory processes^10,11,14–16^.

The primary goal of the present study was to provide more conclusive behavioral evidence to support the SRD hypothesis. To this aim, we compared individuals with aMCI and healthy controls in terms of mind-wandering while performing the task that met all the criteria to capture spontaneous stimulus-dependent retrieval, and included either highly meaningful or unmeaningful pictorial stimuli. Several important findings emerged from this comparison that provide very strong support for the SRD hypothesis.

Most importantly, in line with the SRD hypothesis, individuals with aMCI experienced significantly less spontaneous task-unrelated thoughts than HC. Second, we demonstrated the robustness of the spontaneous retrieval deficits by showing that, for the first time, for pictorial material. Third, the present study is the first to demonstrate that the quality of stimuli in the environment, which could potentially trigger spontaneous thoughts, impacts the size of the spontaneous retrieval deficits. This finding unequivocally supports the claim that the deficits involve spontaneous, but the bottom-up and cue-driven, processes. As predicted, the reduction in spontaneous task-unrelated thoughts was found with exposure to highly meaningful stimuli, but not to unmeaningful pictures. Finally, in accordance with^28^ and our hypothesis, the deficits were most pronounced for past-oriented, spontaneous, task-unrelated thoughts (involuntary autobiographical memories). For such thoughts the deficits were significant for both highly meaningful and unmeaningful stimuli, but varied in size: they were much larger for highly meaningful pictures.

Unexpectedly, we found an increase in the number of spontaneous task-unrelated thoughts in aMCI individuals for unmeaningful stimuli, as compared with highly meaningful pictures. When only past-oriented, spontaneous, task-unrelated thoughts were taken into account, mind-wandering no longer significantly differed after the exposure of aMCI participants to unmeaningful stimuli versus highly meaningful stimuli. This suggests that present-oriented thoughts may have been primarily responsible for this unexpected increase (future-oriented thoughts were scarce). Such interpretation is supported by the fact that the type of stimuli did not influence the number of present-oriented, spontaneous, task-unrelated thoughts, either in the between-groups or within-groups comparisons. This explanation is also in line with the studies showing that, in the absence of meaningful stimuli, people tend to experience primarily future- and present-oriented thoughts^41,42^ in which the deficits are less pronounced compared to involuntary autobiographical memories (see^28^ and the present findings). Interestingly, although future-oriented thoughts were much less frequent than present-oriented thoughts in the present study, it is future-oriented thoughts that demonstrated the reduction of mind-wandering in aMCI, albeit a much smaller reduction than that for autobiographical memories. This finding is in line with the results of both neuroimaging research^43–45^ and behavioral studies^46–49^ showing that past-oriented and future-oriented thoughts are based on overlapping cognitive processes. These studies suggest that future-oriented and past-oriented thoughts can be considered two aspects of the same phenomenon, i.e., mental time travel which is the ability to mentally re-experience autobiographical events and pre-experience possible future occurrences^50^.

A possible limitation of our study was using captioned pictures in the Man-made/Natural Task, rather than pictures alone. Although thought probes explicitly asked participants whether they had any picture-related thoughts, and no participant mentioned captions, participants might have difficulties in distinguishing between caption-induced thoughts and picture-induced thinking. This limitation does not change the fact that we extended behavioral data in support of reduced mind-wandering in aMCI to the type of stimuli that had not been used in previous supportive studies. However, it may lead to a slightly different theoretical interpretation of this reduction. If thoughts were caption-induced, then semantic-to-autobiographical memory priming may have been involved^51,52^. This priming takes place when processing semantic information (prime) leads to activation of relevant autobiographical knowledge structures which increases the likelihood of evoking related memories. Mace et al.^51^ consider semantic-to autobiographical priming a specific type of associative priming, occurring between two separate memory systems (semantic and autobiographical). It has been demonstrated in relation to involuntary autobiographical memories, and interestingly, all primed memories were associated only with high-frequency prime words^51,52^. Mace et al.^51,52^ suggest that low-frequency prime words are weakly associated with participants’ personal experience, and therefore may activate very few autobiographical memories. It is likely that, in the present study, caption words for unmeaningful objects were lower in frequency, compared to captions for highly meaningful objects. Therefore, it may be argued that the reduced number of involuntary autobiographical memories in the MCI group was due to impaired spreading of activation between semantic representations of verbal primes and related autobiographical memories. It may be further argued that low-frequency caption words, corresponding to unmeaningful objects, were less able to demonstrate this impairment because they had equally poor associations with participants’ personal experience in both aMCI group and healthy older adults. Future research may test this theoretical interpretation of differences between aMCI and healthy ageing. However, it should be noted that even this alternative interpretation puts emphasis on those deficits in aMCI that are related to automatic/spontaneous processes in memory.

As for practice, our findings may help researchers to develop new and simple cognitive tests to assess spontaneous, stimulus-driven processes, which may be used clinically for detecting early cognitive deterioration and predicting the conversion to AD. In addition to meeting the criteria listed in the Introduction, e.g., undemanding ongoing task, thought probes, distinguishing between spontaneous and deliberate thoughts, such tests should provide patients with highly meaningful environmental stimuli. Our findings suggest that, for healthy older adults, in contrast to individuals with aMCI, such an environment stimulates spontaneous task-unrelated thoughts in general, and past-related thoughts in particular.

## Method

### Participants

A total of 27 healthy older adults and 27 aMCI participants were recruited. To ensure sufficient power, we performed the a priori power analysis on GPOWER 3.1^53^. The effect size calculation was based on mind-wandering reported by Niedźwieńska and Kvavilashvili^28^ (f = 0.718). With an alpha level of 0.05 and the minimum power of 0.95, 28 participants were necessary to find a statistically significant effect in the model. However, in the study of Maillet and Schacter^6^, in which the Man-made/Natural Task was originally used to compare young adults with healthy older adults, older adults reported much more “no thoughts” trials (~ 20%), compared to the task used in the reference study of Niedźwieńska and Kvavilashvili (6%)^28^. This suggested that the Maillet and Schacter task might have been less powerful in eliciting mind-wandering. Although the substantially modified version of the Maillet and Schacter task was used in the present study, to avoid the risk of not having enough power to capture the difference in mind-wandering between aMCI individuals and HC, we recruited about twice as many participants as calculations indicated were necessary.

All participants were recruited from among inhabitants of local nursery houses and members of senior social clubs. All research was performed in accordance with the Declaration of Helsinki. The study was approved by Psychology Research Ethics Committee at the Jagiellonian University. Participants provided written informed consent to take part in the study. For all participants, exclusion criteria included: (a) head/brain injuries, (b) history of cerebrovascular disease, (c) current alcohol or substance dependence, (d) medical, neurological, or psychiatric disorders resulting in cognitive dysfunctions, (e) age less than 65 years. Fluency in Polish and adequate vision and hearing were also required. Exclusion criteria were assessed in the initial interview screening. Participants who passed the screening, completed a battery of experimental and standardized neuropsychological tests.

#### aMCI participants

Participants were assigned to the clinical group using the inclusion criteria that satisfied the diagnostic criteria of aMCI^54,55^ (a) presence of a subjective memory complaint; (b) objective memory impairment evidenced by a score at or below 1.5 *SD* of the mean of age-matched peers on at least one test of the neuropsychological screening battery assessing episodic memory (see the Neuropsychological evaluation section); (c) not meeting the Diagnostic and Statistical Manual of Mental Disorders’ (DSM-5) criteria for dementia (American Psychiatric Association, 2013), (d) preserved general cognitive function as confirmed by a normal score on the Mini-Mental State Examination (MMSE)^56^ (normality cut-off score: 24)^57^; (e) maintained activities of daily living or slight impairment in instrumental activities of daily living, as confirmed by no more than one item showing deterioration in the Instrumental Activities of Daily Living (IADL) subscale of Nurses' Observation Scale for Geriatric Patients (NOSGER)^58,59^; (f) absence of severe depression, as confirmed by a score below 10 on the Geriatric Depression Scale 15^60^.

#### Healthy controls (HC)

Inclusion criteria for the HC group were: (a) a score within or above 1.5 *SD* of the mean of age-matched peers on each test of the neuropsychological screening battery assessing episodic memory; (c) a score ≥ 27 on the MMSE; (d) no impairment in instrumental activities of daily living, as confirmed by minimum score in the Instrumental Activities of Daily Living (IADL) subscale of Nurses' Observation Scale for Geriatric Patients (NOSGER)^58,59^; (e) absence of severe depression, as confirmed by a score of below 10 on the GDS 15.

Table 2 shows demographic details of the final sample. A series of independent samples t-tests revealed no significant group differences between aMCI and HC on the demographic variables, except for MMSE scores, which were lower in aMCI individuals than in HC (*p* = *0.0*00; *d* = 1.74).

**Table 2:** Demographic characteristics as a function of group (aMCI vs HC).

	aMCI (n = 27)	HC (n = 27)
Sex	10 males	10 males
Age (SD)	79.44 (8.18)	77.77 (7.71)
Years of education (SD)	11.66 (2.54)	12.68 (3.10)
Health at present (SD)	3.14 (0.86)	3.37 (1.00)
Health compared to peers (SD)	3.70 (0.95)	3.59 (0.84)
GDS	3.85 (2.82)	3.07 (1.66)
MMSE	26.59 (1.18)***	28.59 (1.11)

Health at present (1 = poor, 5 = excellent); Health compared to peers (1 = significantly worse, 3 = same, 5 = significantly better).

*aMCI* amnestic Mild Cognitive Impairment, *HC* Healthy Controls.

Differences between aMCI and HC are indicated by *** *p* < .001.

### Measures

#### Neuropsychological evaluation

The episodic memory tests included the Hopkins Verbal Learning Test **(**HVLT)^61,62^ and California Verbal Learning Test (CVLT)^63,64^. HVLT consists of three immediate recall tests, one delayed recall test and one delayed recognition test. CVLT includes five immediate recall tests, one short delay recall test, one long delay recall test and one delayed recognition test. Attention, executive functions, language and visuospatial abilities were tested with the Addenbrooke’s Cognitive Examination-III (ACE-III)^65^. Significant group differences were obtained for all neuropsychological tests, with aMCI participants scoring lower (see Table 3). The effect sizes for episodic memory tests were markedly higher than for the tests measuring other cognitive functions i.e. attention, language, fluency and visuospatial.

**Table 3:** Mean scores on neuropsychological test battery in participants with aMCI and healthy controls.

	aMCI (n = 27)	HC (n = 27)	*d*
**Episodic memory**			
CVLT: immediate recall 1–5	31.55 (9.64)***	45.11 (8.56)	1.48
CVLT: short delay recall	5.51 (2.62)***	9.18 (2.70)	1.40
CVLT: long delay recall	5.18 (2.14)***	10.18 (2.40)	2.19
CVLT: recognition	13.88(2.24)*	15.00 (1.10)	0.63
HVLT: immediate recall 1	3.07 (1.688)**	5.33 (1.41)	1.45
HVLT: immediate recall 2	5.59 (1.96)***	7.37 (1.59)	0.99
HVLT: immediate recall 3	6.11 (1.80)***	8.14 (1.91)	1.09
HVLT: delayed recall	3.29 (2.35)***	6.77 (2.62)	1.38
HVLT: recognition	9.88 (1.88)**	11.03 (0.93)	0.77
**Other cognitive functions**			
ACE-III: attention	16.22 (1.52)*	17.11 (0.97)	0.69
ACE-III: fluency	8.29 (2.65)***	11.12 (1.93)	1.22
ACE-III: language	22.12 (4.46)**	25 (1.41)	0.87
ACE-III: visuospatial	13.48 (1.92)*	14.62 (1.36)	0.68

For each test, a high score indicates a better performance.

*aMCI* amnestic Mild Cognitive Impairment, *HC* healthy controls, *CVLT* California Verbal Learning Test, *HVLT* Hopkins Verbal Learning Test.

Differences between aMCI and HC are indicated by *p < 0.05, **p < 0.01, ***p < 0.001.

#### Mind-wandering evaluation

Participants completed a computer-based Man-made/Natural Task, which was a modified version of the task developed by Maillet and Schacter^6^. The task consisted of a 242-slide presentation of pictures showing natural objects (e.g., flower) and man-made objects (e.g., car). Below each picture there was a caption corresponding to it**.** Participants were asked to decide whether the depicted object was artificial or natural. Each stimulus was presented for 4 s, followed by a blank screen for 4 s. Every 6–10 stimulus slides, the task stopped and thought probe questions appeared on the screen. Participants were asked to describe their thought content the moment before the question appeared on the screen by choosing one of the following answers: (a) I did not have any thoughts; (b) I had a thought triggered by one of the pictures I saw; (c) I had a thought unrelated to the task or any of the pictures I saw; (d) I was thinking how I feel about doing this task. If participants had stimuli-related thoughts, they were additionally asked which picture had triggered the thought. Participants were then asked whether the thought they had was spontaneous or deliberate. Finally, they were asked whether the thought they had were about the past, present or the future. The categories of thoughts and the thought probing procedure were adapted from Maillet and Schacter^6^ (see also^28,32^ for similar thought probing). Thought probes were presented 1.5 s after preceding stimulus slides, since the results of Maillet and Schacter ^6^ suggest that such interval slightly increases the probability of evoking stimulus-related thoughts in healthy adults**.**

Stimulus presentation and the response collection were controlled by Inquisit 5 software running on a 14″ foldable notebook. Pictures measured on average 600 px (height) × 600 px (width) at a viewing distance of 60 cm, and were presented on a white background in the center of the screen. They were generated in a random order, which was then the same for each participant. To simplify the recording of thought probes for Polish older adults who may not be very familiar with using the computer, all participants were giving their answers orally, rather than typing them into the computer as in Maillet and Schacter^6^. The experimenter manually recorded participants’ responses.

We developed two versions of the Man-made/Natural Task. All participants completed the two versions in one session, in a counter-balanced order, without any break between the versions. Each version consisted of 121 blocked pictures of either very familiar objects (the block with highly meaningful stimuli) or unfamiliar objects (the block with unmeaningful stimuli). There were 15 thought probes in each block.

Stimuli-pictures were obtained from the same base as used by Maillet and Schacter^6^, i.e., Bank of Standardized Stimuli^66,67^. The base consists of stimuli that were assessed on different dimensions by a high number of participants, as part of normalization studies. One of these dimensions was familiarity, which was measured with the question: “Rate the level to which you are familiar with the object” on a 5-point scale (1 = very unfamiliar; 5 = very familiar).

For the present study, 300 pictures with the highest scores of familiarity and 300 pictures with the lowest scores of familiarity were chosen from the base. To select the pictures that would be most familiar/unfamiliar to Polish older adults, the pilot study was conducted in which 29 Polish older adults of age 60 + (MA = 67.65; SD = 4.60; 9 Males) were asked the same question about familiarity in relation to each picture chosen form the base. A total of 121 pictures with the highest mean familiarity (M = 4.42; SD = 0.20) and 121 pictures with the lowest mean familiarity (M = 2.81; SD = 0.29) were selected for the final set. Due to the predominance of pictures showing man-made objects among the pictures rated as most familiar and most unfamiliar, pictures with natural objects accounted for 1/3 of stimuli in each version of the Man-made/Natural Task.

### Procedure

Participants were tested individually in two sessions, up to 5 weeks apart, with each session lasting approximately one hour. Sessions took place on the premises of the nursing houses and senior social clubs, in quite separate rooms. The screening interview, NOSGER-IADL, MMSE, ACE-III, CVLT and Geriatric Depression Scale 15 were administrated in Session 1. The Man-made/Natural Task and HVLT were completed in Session 2.

At the beginning of Session 2 participants completed the short-delay HVLT tasks. They were then briefly introduced to the Man-made/Natural Task. Participants were asked to press “S” on the keyboard if the object on the screen was man-made, and press “N” if it was natural. They were also informed that we are interested in what types of thoughts people experience while performing such tasks. Therefore, the slide presentation would occasionally stop, at which point they would be prompted to report their thoughts at the exact moment they were stopped. Participants were briefly informed about the thoughts they might experience during the task and what options they would have to categorize them, i.e., no thoughts, picture-triggered off-task thoughts, picture-unrelated off-task thoughts, and thoughts about the experience of performing the task. This was followed by training, during which participants were given examples of thoughts from various categories and asked what category they would choose. If they made the wrong choice, they were explained why it should be a different category. The exemplary thoughts were, among others: *I used to work as a bus-driver* after seeing the picture of a bus; *I have a doctor appointment tomorrow*, with a no picture related to such fact; *I wonder if I have chosen the right answer*. The training continued until the participant was able to correctly categorize all types of thoughts. Participants were then explained the difference between spontaneous thoughts (thoughts that pop into mind without your intention) and deliberate thoughts (something you deliberately chose to think about). Finally, participants were briefly informed about the types of off-task thinking they could experience, i.e., that it could be related to something that: (a) was happening in the present, at any point in the course of the task (e.g., *I love my family*); (b) had happened in the past, before starting the task (e.g., *I went to Spain last year*); (c) would happen in the future, after completing the task (e.g., *I’m going to eat delicious supper today*). This was followed by short practice with two 10-slides trials and two thought probes. After practice, participants completed the long-delay HVLT tasks and then both versions of the Man-made/Natural Task. When the procedure was completed, participants were asked to rate how interesting the task of classifying pictures was (1 = very boring; 10 = very interesting), and how difficult the task of categorizing thoughts was (1 = very difficult; 10 = very easy).

## Supplementary Information

Supplementary Tables.

## Data Availability

The data used to support the findings of this study are available from the corresponding author upon request.
