# Supplementary material for: Deficits in spontaneous and stimulus-dependent retrieval as an early sign of abnormal aging
Source: Sci Rep. 2022 Jun 10;12:9643. doi: 10.1038/s41598-022-13745-6 (PMC9187621; doi:10.1038/s41598-022-13745-6)
Supplement: Supplementary file 1 — Supplementary Tables. [file 41598_2022_13745_MOESM1_ESM.docx]

**Supplementary Materials**

| **Table S1**  *Between-Group Comparisons for each Response Type* | | | | | |
| --- | --- | --- | --- | --- | --- |
| **Response Type** | **Group** | **M (SD)** | **F (1, 52)** | **p** | **η^2^** |
| Spontaneous Task-Unrelated | HC  aMCI | 13.741 (7.60)**  7.85 (8.00) | 7.678 | .008 | .129 |
| Spontaneous Task-Related | HC  aMCI | 6.519 (5.89)  6.741 (8.19) | 0.013 | .909 | .000 |
| Deliberate Thoughts | HC  aMCI | 3.259 (6.00)  4.148 (8.50) | 0.197 | .659 | .004 |
| No Thoughts | HC  aMCI | 6.481 (5.68)*  11.26 (9.16) | 5.299 | .025 | .092 |
| *Note:* aMCI= amnestic Mild Cognitive Impairment; HC = Healthy Controls  Differences between aMCI and HC are indicated by * *p* < .05, ** *p* < .01 | | | | | |

| **Table S2**  *Between-Group Comparisons in the Number of Spontaneous Task-Unrelated Thoughts for each Stimulus Type (Highly Meaningful vs Unmeaningful)* | | | | | |
| --- | --- | --- | --- | --- | --- |
| **Stimulus Type** | **Group** | **M (SD)** | **F (1, 52)** | **p** | **η^2^** |
| Highly Meaningful | HC  aMCI | 7.41 (4.34)***  3.22 (3.73) | 14.412 | .000 | .217 |
| Unmeaningful | HC  aMCI | 6.33 (3.95)  4.63 (4.59) | 2.135 | .150 | .039 |
| *Note:* aMCI= amnestic Mild Cognitive Impairment; HC = Healthy Controls  Differences between aMCI and HC are indicated by *** *p* < .001 | | | | | |

| **Table S3**  *Within-Group Comparisons in the Number of Spontaneous Task-Unrelated Thoughts for each Stimulus Type (Highly Meaningful vs Unmeaningful)* | | | | | |
| --- | --- | --- | --- | --- | --- |
| **Group** | **Stimulus Type** | **M (SD)** | **F (1, 52)** | **p** | **η^2^** |
| HC | Highly Meaningful  Unmeaningful | 7.41 (4.34)  6.33 (3.95) | 3.645 | .062 | .066 |
| aMCI | Highly Meaningful  Unmeaningful | 3.22 (3.73)*  4.63 (4.59) | 6.258 | .016 | .107 |
| *Note:* aMCI= amnestic Mild Cognitive Impairment; HC = Healthy Controls  Differences between aMCI and HC are indicated by * *p* < .05 | | | | | |

| **Table S4**  *Between-group Comparisons in the Number of Spontaneous Task-Unrelated Thoughts for each Temporal Orientation (Present vs Past vs Future)* | | | | | |
| --- | --- | --- | --- | --- | --- |
| **Temporal Orientation** | **Group** | **M (SD)** | **F (1, 52)** | **p** | **η^2^** |
| Present | HC  aMCI | 3.56 (3.36)  4.63 (6.24) | .619 | .435 | .012 |
| Past | HC  aMCI | 8.70 (6.04)***  2.44 (3.56) | 21.282 | .000 | .292 |
| Future | HC  aMCI | 1.44 (1.34)*  0.59 (1.43) | 5.136 | .028 | .090 |
| *Note:* aMCI= amnestic Mild Cognitive Impairment; HC = Healthy Controls  Differences between aMCI and HC are indicated by * *p* < .05, *** *p* < .001 | | | | | |

| **Table S5**  *Between-Group Comparisons in the Number of Past-Oriented Spontaneous Task-Unrelated Thoughts for each Stimulus Type (Highly Meaningful vs Unmeaningful)* | | | | | |
| --- | --- | --- | --- | --- | --- |
| **Stimulus Type** | **group** | **M (SD)** | **F (1, 52)** | **p** | **η^2^** |
| Highly Meaningful | HC  aMCI | 4.70 (3.51)***  0.67 (1.17) | 31.991 | .000 | .381 |
| Unmeaningful | HC  aMCI | 4.00 (3.36)*  1.78 (2.80) | 6.952 | .011 | .118 |
| *Note:* aMCI= amnestic Mild Cognitive Impairment; HC = Healthy Controls  Differences between aMCI and HC are indicated by * *p* < .05, *** *p* < .001 | | | | | |

| **Table S6**  *Within-Group Comparisons in the Number of Past-Oriented Spontaneous Task-Unrelated Thoughts for each Stimulus Type (Highly Meaningful vs Unmeaningful)* | | | | | |
| --- | --- | --- | --- | --- | --- |
| **Group** | **Stimulus Type** | **M (SD)** | **F (1, 52)** | **p** | **η^2^** |
| HC | Highly Meaningful  Unmeaningful | 4.70 (3.51)  4.00 (3.36) | 1.608 | .210 | .030 |
| aMCI | Highly Meaningful  Unmeaningful | 0.67 (1.17)  1.78 (2.80) | 4.010 | .051 | .072 |
| *Note:* aMCI= amnestic Mild Cognitive Impairment; HC = Healthy Controls | | | | | |
